# On TCR binding predictors failing to generalize to unseen peptides

**DOI:** 10.3389/fimmu.2022.1014256

**Published:** 2022-10-21

**Authors:** Filippo Grazioli, Anja Mösch, Pierre Machart, Kai Li, Israa Alqassem, Timothy J. O’Donnell, Martin Renqiang Min

**Affiliations:** ^1^ Biomedical AI Group, NEC Laboratories Europe, Heidelberg, Germany; ^2^ Machine Learning Department, NEC Laboratories America, Princeton, NJ, United States; ^3^ Division of Hematology and Medical Oncology, Icahn School of Medicine at Mount Sinai, New York, NY, United States

**Keywords:** tcr, peptide, MHC, binding prediction, interaction prediction, machine learning, TCR - T cell receptor

## Abstract

Several recent studies investigate TCR-peptide/-pMHC binding prediction using machine learning or deep learning approaches. Many of these methods achieve impressive results on test sets, which include peptide sequences that are also included in the training set. In this work, we investigate how state-of-the-art deep learning models for TCR-peptide/-pMHC binding prediction generalize to unseen peptides. We create a dataset including positive samples from IEDB, VDJdb, McPAS-TCR, and the MIRA set, as well as negative samples from both randomization and 10X Genomics assays. We name this collection of samples *TChard*. We propose the *hard split*, a simple heuristic for training/test split, which ensures that test samples exclusively present peptides that do not belong to the training set. We investigate the effect of different training/test splitting techniques on the models’ test performance, as well as the effect of training and testing the models using mismatched negative samples generated randomly, in addition to the negative samples derived from assays. Our results show that modern deep learning methods fail to generalize to unseen peptides. We provide an explanation why this happens and verify our hypothesis on the *TChard* dataset. We then conclude that robust prediction of TCR recognition is still far for being solved.

## 1 Introduction

Studying T-cell receptors (TCRs) has become an integral part of cancer immunotherapy and human infectious disease research (1–4). TCRs are able to identify intracellular processed peptides originating from infected or aberrant cells. TCRs are heterodimers consisting of an α- and a β-chain, which bind to peptides presented on the cell surface by either major histocompatibility complex (MHC) class I or class II molecules, depending on the cell type (5–7). The binding of the TCR to the peptide-MHC (pMHC) complex occurs primarily (but not exclusively) at the complementarity-determining region 3 (CDR3). The CDR3α consists of alleles from the V and J genes; for the CDR3β, the D gene is additionally involved (8, 9). These alleles can be recombined unboundedly, which results in a high TCR repertoire diversity, essential for a broad T cell-based immune response (10). When a naive TCR is exposed to an antigen and activated for the first time, a memory T-cell population with this TCR may develop, which enables a long-lasting immune response (11, 12).

Numerous recent studies investigate TCR-peptide/-pMHC binding prediction by applying different machine or deep learning methods (13–24). Many of these studies use data from the Immune Epitope Database (IEDB) (25), VDJdb (26), and McPAS-TCR (27), which mainly contain CDR3β data and lack information on CDR3α. Such methods achieve high test performance when evaluated on test sets that belong to the same source as the training set. However, we show that these methods exhibit weak cross-dataset generalization, i.e., the models suffer from severe performance degradation when tested on a different dataset. For example, as shown in **Figure S1**, several machine learning models trained on McPAS-TCR perform poorly on VDJdb.

**Figure 1:**
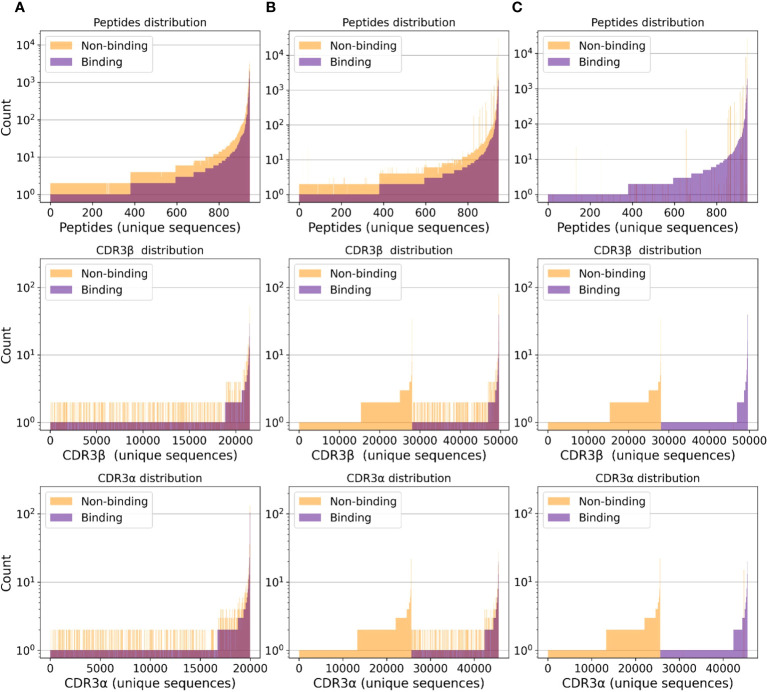
Separate class distributions for unique peptides (first row), CDR3*β* (second row), and CDR3*α* (third row) sequences in all (*peptide, CDR3β, CDR3α*) samples. A point on the ***x***-axis represents one unique sequence of amino acids. The ***y***-axis represents how frequently a given peptide, CDR3*β*, or CDR3*α* sequence appears in the considered samples. Sequences are sorted by count. **(A)** Negative samples only include randomized data points (i.e., no negative assays). **(B)** Negative samples include negative assays and randomized negative samples. **(C)** Negative samples only include negative assays.

In this work, in order to evaluate the relevance of the available data for deep-learning-based TCR-peptide/-pMHC binding prediction, we aggregate binding samples obtained from IEDB, VDJdb, and McPAS-TCR. Non-binding data points are collected from IEDB, as well as from the 10X Genomics samples provided in the NetTCR-2.0 repository (22). We additionally consider a set of samples from (28, 29), which are included in the NetTCR-2.0 GitHub repository; we refer to it as the MIRA set. A simple analysis of the class distribution (binding versus non-binding) of the resulting data points reveals that all TCR sequences exclusively appear in either binding or non-binding TCR-peptide/-pMHC pairs; no CDR3 sequence is observed in both positive and negative samples (see **Figure 1C**). Machine learning models trained naively on data with this class distribution are prone to learning undesirable inductive biases. In fact, our results in *Section 4.1* suggests that they tend to classify samples only as a function of the CDR3 sequences, which could be memorized.

For unbiased evaluation, we perform experiments on a dataset derived from the integration of the aforementioned samples. We name the resulting collection of samples *TChard*. To the best of our knowledge, this dataset constitutes the largest set of TCR-peptide/-pMHC samples available at the time this work is being written.

We perform deep learning experiments using two state-of-the-art models for TCR-peptide/-pMHC interaction prediction: ERGO II (23) and NetTCR-2.0 (22). ERGO II is a deep learning approach that adopts long short-term memory (LSTM) networks and autoencoders to compute representations of peptides and CDR3s. It can also handle additional input modalities, i.e., V and J genes, MHC, and T-cell type. NetTCR-2.0 employs a simple 1D CNN-based model, integrating peptide and CDR3 sequence information for the prediction of TCR-peptide specificity. Both models input peptide and CDR3s representations in the form of amino acid sequences. The selection of these two models is motivated by the intention to analyze two of the most successful classes of deep learning models: feed-forward convolutional networks (e.g., NetTCR-2.0) and recurrent neural networks (e.g., ERGO II, which includes an LSTM encoder). For this analysis, we do not consider methods that rely on external source of information, e.g., TITAN [24], which performs pre-training on BindingDB (30).

We perform experiments on *TChard* and investigate the effect of different training/test splitting strategies. In contrast to previous works (22, 23), we place special emphasis on testing the models on unseen peptides. We propose the *hard split*, a splitting heuristic meant to create test sets that only contain unseen peptides, i.e., not included in the training set. In the context of neoantigen-based cancer vaccines development, neoepitopes exhibit enormous variability in their amino acids sequences; employing TCR binding predictors for this application requires robust generalization to unseen peptides. In accordance with recent findings (17), we show that evaluating the models’ performance on unseen peptides leads to poor generalization. In the **Supplementary Material**, we describe the training/test splitting strategies adopted by Montemurro et al. (22) and Springer et al. (23).

## 2 The *TChard* dataset

In this section, we describe the creation of the *TChard* dataset. All samples in *TChard* include a peptide and a CDR3β sequence, associated with a binary binding label. A subset of these samples may additionally have (i) CDR3a sequence information, and/or (ii) allele information of the MHC (class I or II) in complex with peptides. A sample consists therefore of a tuple of molecules (from 2 to 4). When available, the V and J alleles for the α-chain and the V, D, and J alleles for the β-chain are also included. We refer to the binding tuples as *positive* and to the non-binding ones as *negative*.

### 2.1 Dataset creation

First, we collect positive assays from the IEDB, VDJdb^1^, and McPAS-TCR databases. Additionally, we include the binding samples from the MIRA set (28, 29), which are publicly available in the NetTCR-2.0 repository^^2^^.

Second, we include negative assays, i.e., non-binding tuples of molecules extracted from IEDB. Additionally, a set of negative samples extracted from the NetTCR-2.0 repository is considered; this is derived from 10X Genomics assays described by Montemurro et al. (22). In this work, we refer to the negative tuples derived from negative assays as the NA set.

Third, we operate a filtration over the length of the amino acid sequences, and we only keep samples with peptide sequence length smaller than 16, CDR3α sequence length between 7 and 21, and CDR3β sequence length between 9 and 23. These filtration steps are meant to exclude a small portion of data points that present consistently longer amino acid sequences. Including them in the dataset would imply extending the magnitude of the padding required by NetTCR-2.0 by a large margin, making computation more expensive.

Fourth, we generate negative samples *via* random recombination of the sequences found in the positive tuples. Building from the positive samples, we associate the peptides or pMHC complexes (when MHC allele information is available) with CDR3α and CDR3β sequences randomly sampled from the dataset, as operated in previous studies (23). We sample twice as many mismatched negative samples as there are positive ones. We discard randomly generated samples that share at least the same (peptide, CDR3β) with any positive sample. In this work, we refer to the randomized negative tuples as the RN set. Additional remarks on invalid residues and CDR3 sequence homogenization are included in the **Supplementary Material**.

### 2.2 Description of the data distributions

The full dataset, i.e., considering negative samples from both NA and RN, presents the following:

• 528,020 unique (*peptide, CDR3β*) tuples, 385,776 of which are negative and 142,244 are positive;
• 400,397 unique (*peptide, CDR3β, MHC*) tuples, 300,168 of which are negative and 100,229 are positive;
• 111,041 unique (*peptide, CDR3β, CDR3α*) tuples, 82,631 of which are negative and 28,410 are positive; and
• 110,266 unique (*peptide, CDR3β, CDR3α, MHC*) tuples, 82,037 of which are negative and 28,229 are positive.

The dataset statistics considering negative samples derived from either RN or NA are presented in **Table S1**. **Figure 1** depicts the class distribution for (*peptide, CDR3β, CDR3α*) samples. Analogously, **Figures S2–S4** depict the class distribution for (*peptide, CDR3β*), (*peptide, CDR3β, MHC*) and (*peptide, CDR3β, CDR3α, MHC*) samples, respectively. **Figure S5** depicts the length distribution for all sequences.

## 3 Predicting TCR recognition with deep learning

We perform experiments on the *TChard* dataset with two publicly available state-of-the-art deep learning methods for TCR-peptide/-pMHC interaction prediction: ERGO II and NetTCR-2.0 ^^3^^.

We operate TCR-peptide interaction prediction considering peptide and CDR3β, as well as TCR-pMHC interaction prediction considering peptide, CDR3β, CDR3α, and MHC. NetTCR-2.0 is not explicitly designed to account for MHC information; we circumvent this shortcoming by concatenating the MHC pseudo-sequence ^4^ to the other input amino acid sequences and perform BLOSUM50 encoding (32). We do not make distinctions between class I and II MHCs and train a single model for both types.

### 3.1 Random and hard training/test splits

For performance evaluation, we investigate two different strategies for training/test splits.

**Random split (RS).** Given a training/test ratio (80/20 in this work), this procedure consists in sampling test samples uniformly from the dataset without replacement until the desired budget is filled. The remaining samples constitute the training set. In this work, we refer to RS(RN), when the negative tuples only belong to the RN set, to RS(NA), when the negative tuples only belong to the NA set, and to RS(RN+NA), when all negative samples are considered.

The nature of TCR recognition is combinatorial. In our dataset, although a given tuple of molecules is only observed once, a given peptide can appear multiple times, paired with different CDR3β, CDR3α, or MHC. Using a random training/test split ensures that test tuples are not observed at training time. However, this can lead to testing the model on peptides, MHCs, or CDR3β and CDR3α sequences that were already observed at training time in combination with different sequences. Our results show that this can lead to overoptimistic estimates of machine learning models’ real-world performance. To enable neoantigen-based cancer vaccines and T-cell herapy, it is fundamental to test the model on sequences that were never observed at training time. Neoantigens display in fact enormous variability in their amino acids sequence; to identify the most immunogenic vaccine elements, we need models that generalize to unseen sequences.

**Hard split (HS).** We propose a simple heuristic, which we refer to as *hard split*. Considering the whole dataset consisting in a set of tuples, we first select a *minimum* training/test ratio (85/15 in this work). Let *P_l,u_* be the set of all peptides that are observed in at least *l* tuples but no more than *u* tuples in our dataset. We randomly sample a peptide from *P_l,u_* without replacement. All tuples that include that peptide are assigned to the test set. If the current number of test samples is smaller than the budget defined by the training/test ratio, the sampling from *P_l,u_* is repeated.

This heuristic ensures that the peptides that belong to the test set are not observed by the model at training time. For the (*peptide, CDR3β*) tuples, which present 1,360 different peptides, we set *l* and *u* to 500 and 10,000, respectively. This selects a set of 104 possible test peptides. For the (*peptide, CDR3β, CDR3α, MHC*) tuples, which present 870 different peptides, we set *l* and *u* to 100 and 5,000, respectively. This results in a set of 42 possible test peptides. The *l* parameter is a lower bound and ensures that the selected test peptides are paired with a sufficiently broad variety of CDR3 sequences. The *u* parameter is an upper bound and allows excluding test peptides that can too quickly saturate the test budget, hence reducing the variety of test peptides. We create five different hard splits using five different random seeds for the sampling of the test peptides. For the creation of the hard training/test splits, we consider all positive samples, as well as the negative samples from the RN set, i.e., excluding the negative samples from the negative assays. We refer to this type of split as HS(RN).

**Tables S2** and **S3** describe the different HS(RN) hard splits for the (*peptide, CDR3β*) and (*peptide, CDR3β, CDR3α, MHC*) samples, respectively. They present the lists of test peptides and the number of positive and negative samples associated with each of them. Each displayed test is paired with different TCRs. The test TCRs can be observed at training time, as the HS only ensures that test peptides are unseen.

Since a subset of the available samples is included in more than one source database, we drop duplicate data points for the two considered settings, i.e., (*peptide, CDR3β, label*) and (*peptide, CDR3β, CDR3α, MHC, label*).

### 3.2 Validation approach and performance evaluation

For robust performance evaluation, we repeat the experiments for each different training/test split (i.e., five times). The area under the receiver operator characteristic (AUROC) curve (33, 34), the area under the precision–recall (AUPR) curve (35, 36), the F1 score (F1) (37), and precision, recall, and classification accuracy are computed on the test sets and averaged.

We adopt the default configuration for both ERGO II and NetTCR-2.0, as proposed in their original implementations. For ERGO II, we adopt the LSTM amino acid sequences encoder. The training is performed for a maximum of 1,000 epochs and, in order to avoid over-fitting, the best model is selected by saving the weights corresponding to the epoch where the AUROC is maximum on the validation set. The validation set is obtained *via* 80/20 stratified random split of the training set.

## 4 Results

**Figure 2:**
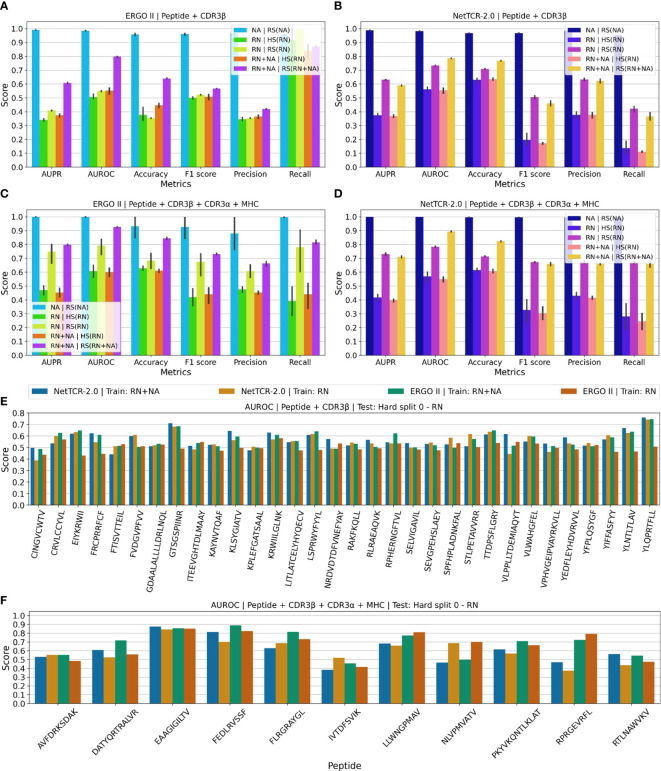
Test results for ERGO II and NetTCR-2.0 for TCR-peptide/-pMHC interaction prediction trained and tested on *TChard*. AUPR: area under the precision–recall curve. AUROC: area under the receiver operator characteristic curve. NA: negative samples from negative assays. RN: negative samples from random mismatching. RS(·): random split. HS(·): hard split. Confidence intervals are standard deviation over 5 experiments with independent training/test splits. **(A–D)** ERGO II and NetTCR-2.0 results on (*peptide, CDR3β*) and (*peptide, CDR3β, CDR3α, MHC*) samples. Legend: *Source of training negatives | Training/test split*. **(E)** Peptide-specific AUROC computed on the (*peptide, CDR3β*) test set obtained with hard split 0 (see **Table S2**). **(F)** Peptide-specific AUROC computed on the (*peptide, CDR3β, CDR3α, MHC*) test set obtained with hard split 0 (see Table S3).

**Figure 2** shows test results for ERGO II and NetTCR-2.0, for the RS and HS splitting strategies, in both the peptide+CDR3β and the peptide+CDR3β+CDR3α+MHC settings. We perform experiments considering negative samples from the NA set only, from the RN set only, and jointly from both the NA and RN sets. Additionally, in the **Supplementary Material**, we report results of experiments performed exclusively on VDJdb samples with quality score ≥ 1.

### 4.1 Overoptimistic classification performance due to sequence memorization

As depicted in **Figure 2**, almost perfect classification is achieved when training with negative samples only from the NA set and testing using the RS(NA) split. As shown in **Figures S2C** and **S4C**, when considering negative samples from the NA set only, the binding and non-binding class histograms of the CDR3 sequences are disjoint. Hence, models can learn to correctly map a large portion of test tuples to the correct label simply by memorizing the CDR3 sequences, ignoring the peptide. We believe that these results are overoptimistic and should not be considered as the approximation of these models’ real-world performance.

### 4.2 The hard split allows for realistic evaluation

Using the HS heuristic appears to make prediction on the test set consistently harder, if not impossible. This tendency is observed in the peptide+CDR3β setting (**Figures 2A, B**) and in the peptide+CDR3β+CDR3α+MHC setting (**Figures 2C, D**). In the peptide+CDR3β setting, when testing the models using the HS(RN) split, the predictions on the test set barely exceed random-level performance, i.e., almost no generalization to unseen peptides is occurring (AUROC ≈ 0.55). This phenomenon is observed when the models are trained using negative samples from the RN set only, as well as when using negative samples from both the RN and NA sets.

The effect of including negative samples from NA at training time does not significantly influence test performance when the HS is adopted. Conversely, when RS is performed, significant differences are caused by the utilization of the negative samples from NA. This reinforces our claims regarding sequence memorization. ERGO II, in the peptide+CDR3β setting (**Figure 2A**), achieves overoptimistic performance when the negative samples come from both NA and RN and testing is operated using RS(RN+NA). The same phenomenon is observed in **Figure 2B** for ERGO II in the peptide+CDR3β+CDR3α+MHC setting and in **Figure 2D** for NetTCR-2.0 in the peptide+CDR3β+CDR3α+MHC setting.

**Figure S6** depicts NetTCR-2.0 results on the (*peptide, CDR3β, CDR3α, MHC*) samples, but ignoring the MHC; we report these results for fairness, as NetTCR-2.0 is not originally designed to handle MHC pseudo-sequences.

## 5 Discussion

In this work, we aim to test the reliability of state-of-the-art deep learning methods on TCR-peptide/-pMHC binding prediction for unseen peptides. To this purpose, we integrate TCR-peptide/-pMHC samples from different databases. We name this collection of samples *TChard*.

We perform experiments with two state-of-the-art deep learning models for TCR-peptide/-pMHC interaction prediction, ERGO II and NetTCR-2.0. We study the peptide+CDR3β and the peptide+CDR3β+CDR3α+MHC settings. We compare the effect of different training/test splitting strategies, RS and HS. RS is a naive random split, while HS allows testing the models on unseen peptides. We investigate the effect of training and testing the models using mismatched negative samples generated randomly (RN), in addition to the negative samples derived from assays (NA).

As shown in our experiments, when the HS is performed, the two models do not generalize to unseen peptides; this appears to be in contrast to the TPP-III results presented by Springer et al. (23). Conversely, when a simple RS is employed and negative samples only belong to NA, almost perfect classification is achieved. We believe that this phenomenon is due to the class distribution of the CDR3 sequences and the related sequence memorization. As shown in **Figure 1C**, when considering negative samples from NA only, the positive and the negative samples are completely disjoint. Hence, a given CDR3 sequence is only presented in either binding or non-binding samples. This leads to learning an inductive bias, which classifies tuples as binding or non-binding exclusively based on the CDR3 sequence, without considering which peptide they are paired with; this appears to be confirmed also by the findings of Weber et al. (24).

In order to make progress towards robust TCR-peptide/-pMHC interaction prediction, machine learning models should achieve satisfactory test performance on the hard training/test split (HS), which we propose in this work. Only then will such models be applicable for real-world applications, e.g., personalized cancer immunotherapy and T-cell engineering. Possible strategies to achieve this goal might require exploring different feature representations, e.g., SMILES (38) encodings as proposed in TITAN (24). Further possible methods might rely on physics-based simulations for the generation of large-scale datasets. Additionally, transfer learning techniques (39) might allow to leverage knowledge from large databases of protein-ligand binding affinity, e.g., BindingDB (30), which includes more than 1 million labeled samples.

## Data availability statement

The dataset adopted for this study can be found in the following repository: https://doi.org/10.5281/zenodo.6962043. The code used to create the dataset and to run the machine learning experiments can be found in https://github.com/nec-research/tc-hard.

## Author contributions

FG pre-processed the data, created the dataset, performed the machine learning experiments, and drafted the manuscript. All other authors contributed to the conceptualization of the work and revised the manuscript. In particular, AM supported the data pre-processing and provided immuno-oncological guidance. All authors contributed to the article and approved the submitted version.

## Footnotes

1 As we aim at creating the largest possible collection of samples, we do not perform any filtration on the quality score of the VDJdb samples, at the cost of introducing noise.

2
https://github.com/mnielLab/NetTCR-2.0/tree/main/data.

3 ERGO II: https://github.com/IdoSpringer/ERGO-II; NetTCR-2.0: https://github.com/mnielLab/NetTCR-2.0.

4 Taken from the PUFFIN [31] repository: https://github.com/gifford-lab/PUFFIN/blob/master/data/.
