# Supplementary material for: On TCR binding predictors failing to generalize to unseen peptides
Source: Front Immunol. 2022 Oct 21;13:1014256. doi: 10.3389/fimmu.2022.1014256 (PMC9634250; doi:10.3389/fimmu.2022.1014256)
Supplement: Supplementary file 1 [file DataSheet_1.pdf]

Supplementary Material

1 SUPPLEMENTARY TABLES AND FIGURES

1.1 Figures

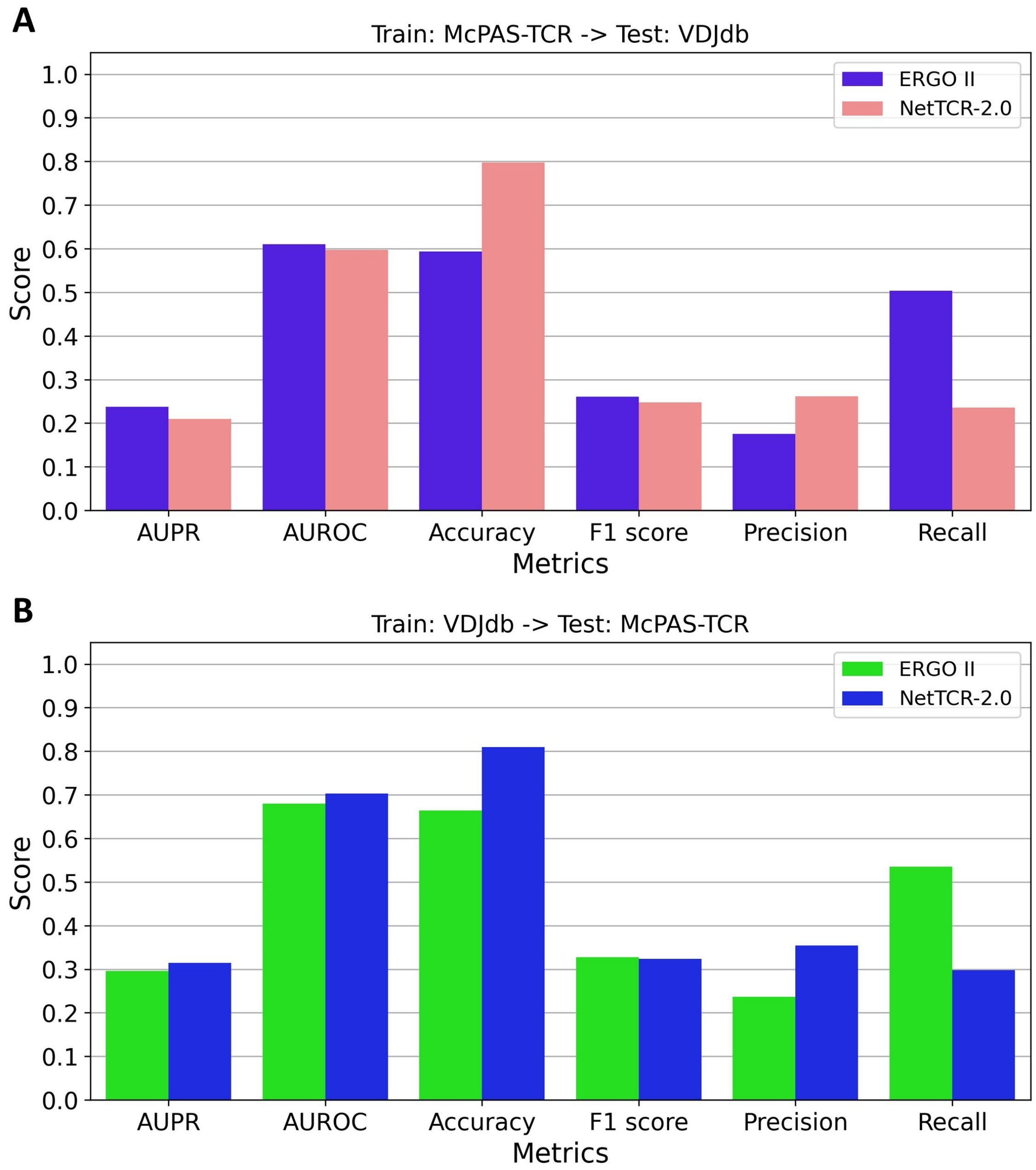

Figure S1: Cross-dataset generalization experiments on $(peptide, CDR3\beta)$ samples. The VDJdb and McPAS-TCR samples provided in the ERGO II GitHub repository have been adopted. As described in Springer et al., negative samples are obtained via random mismatching. AUPR: area under the precision-recall curve. AUROC: area under the receiver operator characteristic curve. **(A)** Models trained on McPAS-TCR and tested on VDJdb. **(B)** Models trained on VDJdb and tested on McPAS-TCR.

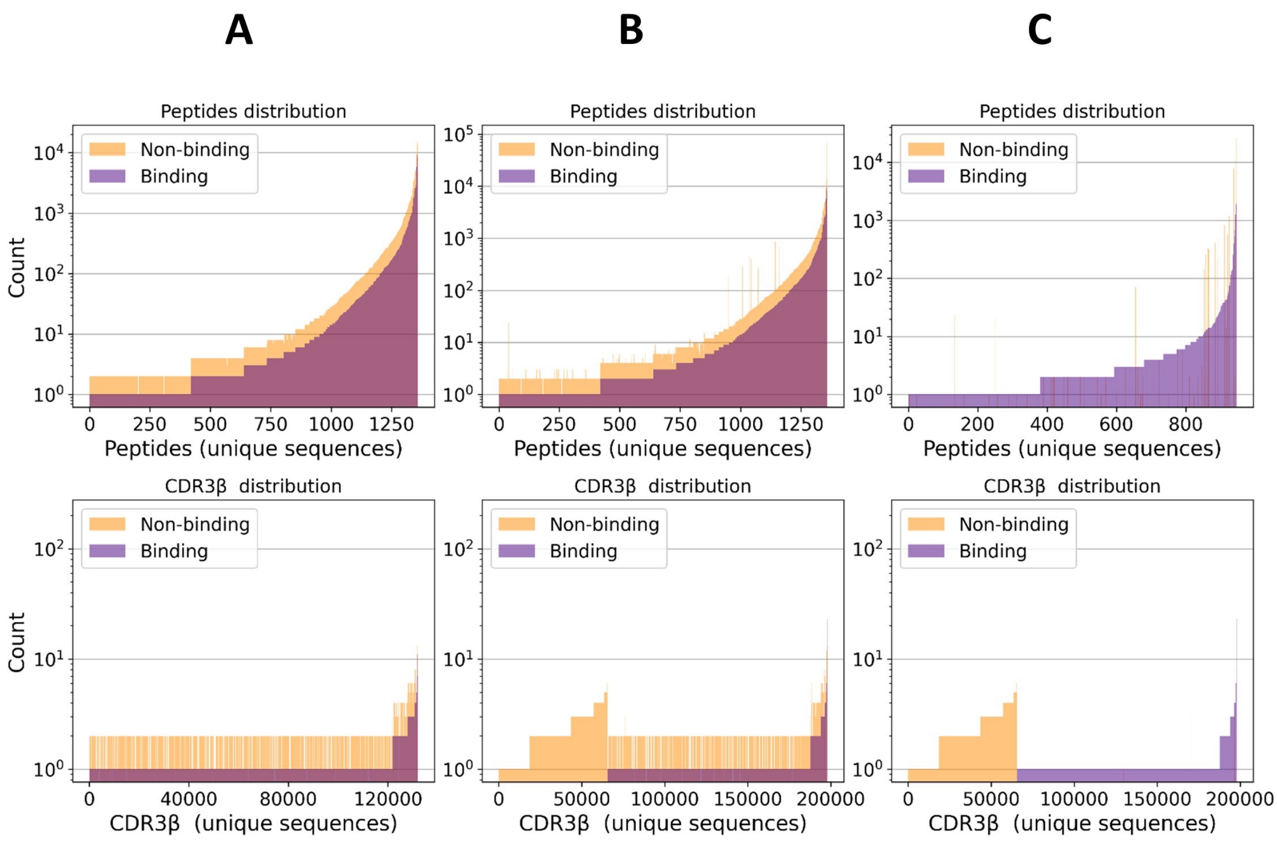

Figure S2: Class distribution of $(peptide, CDR3\beta)$ samples. **(A)** Negative samples only include randomized negative samples (i.e. no negative assays). **(B)** Negative samples include negative assays and randomized negative samples. **(C)** Negative samples only include negative assays.

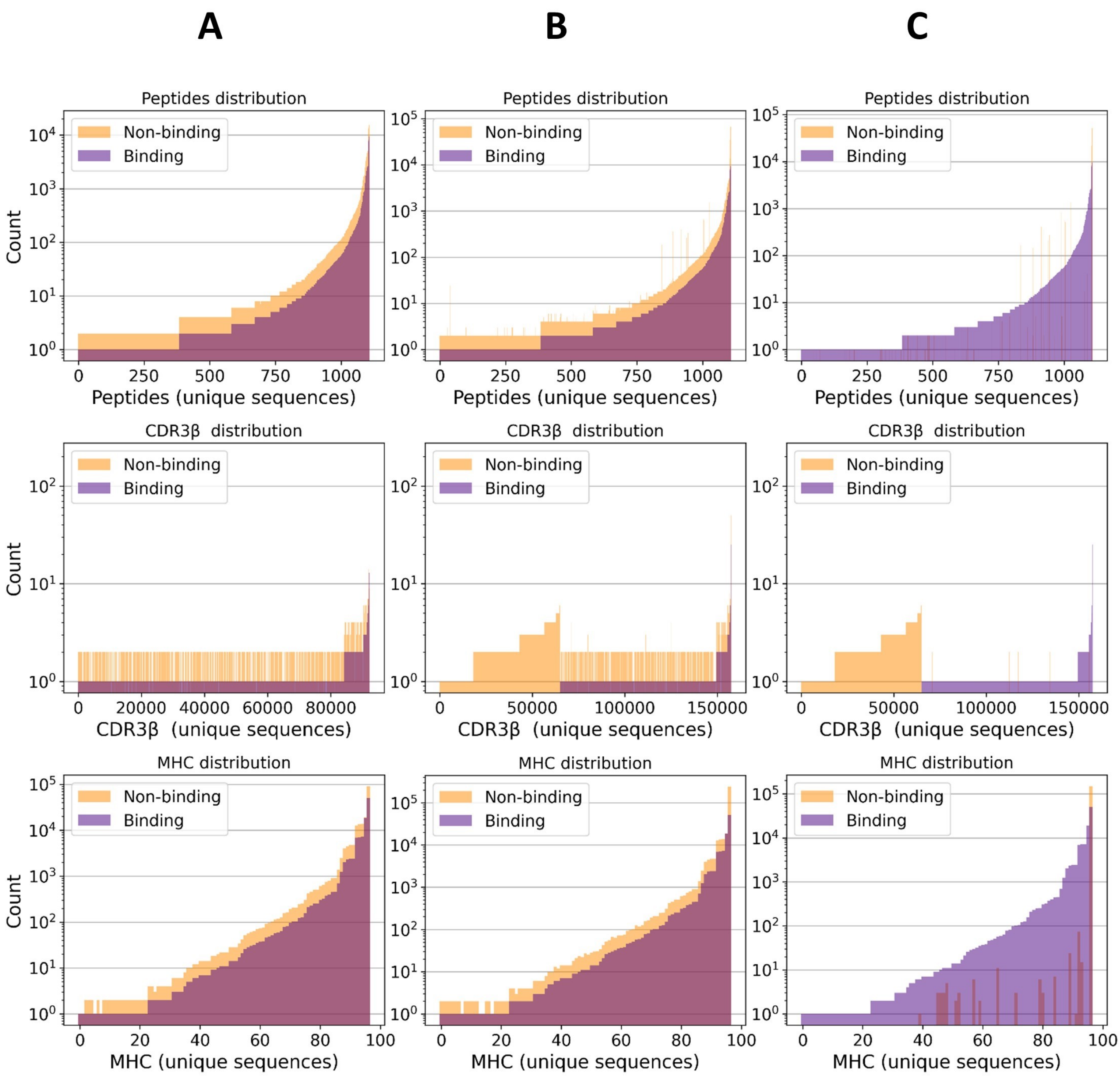

Figure S3: Class distribution of $(peptide, CDR3\beta, MHC)$ samples. **(A)** Negative samples only include randomized negative samples (i.e. no negative assays). **(B)** Negative samples include negative assays and randomized negative samples. **(C)** Negative samples only include negative assays.

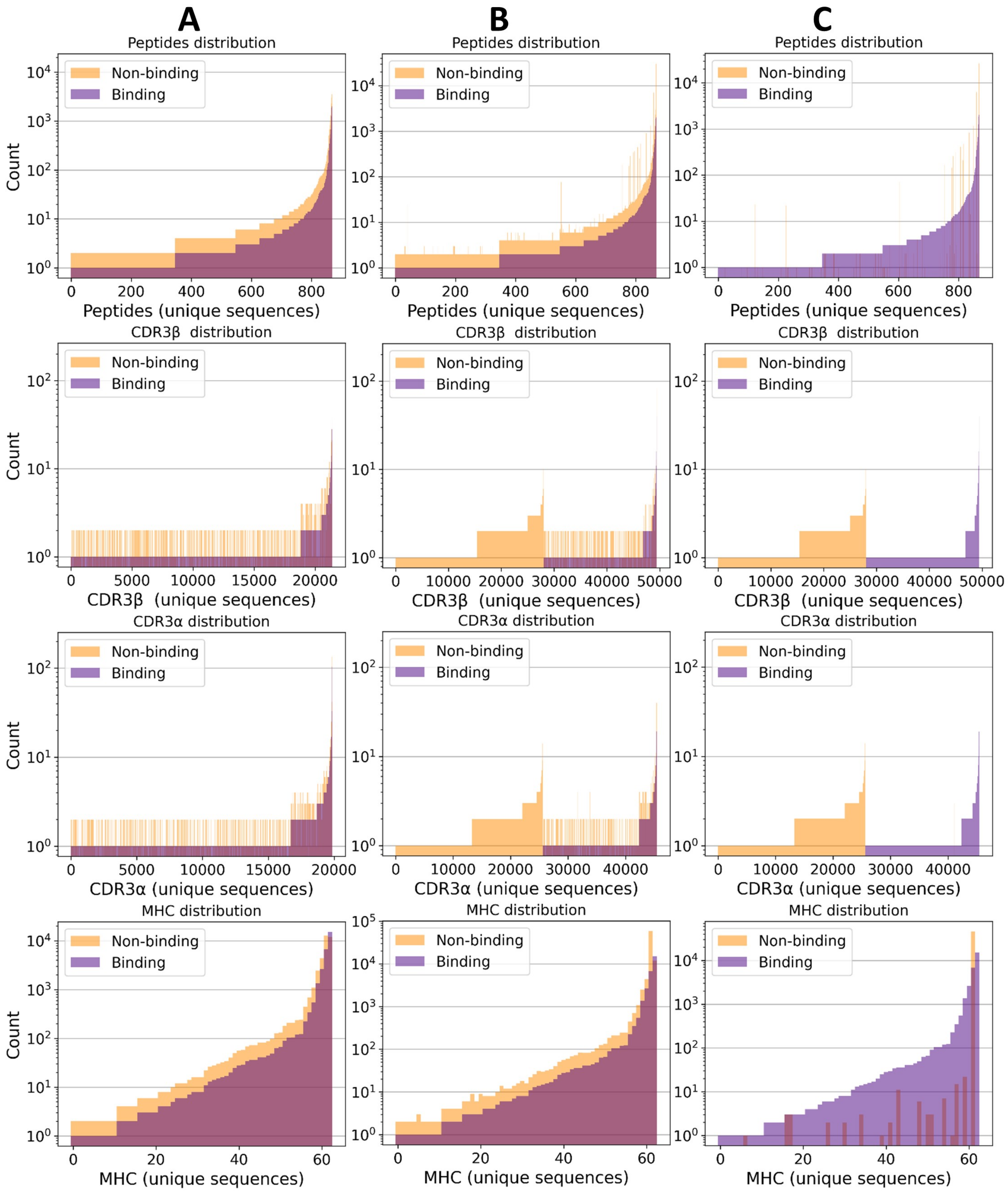

Figure S4: Class distribution of $(peptide, CDR3\beta, CDR3\alpha, MHC)$ samples. **(A)** Negative samples only include randomized negative samples (i.e. no negative assays). **(B)** Negative samples include negative assays and randomized negative samples. **(C)** Negative samples only include negative assays.

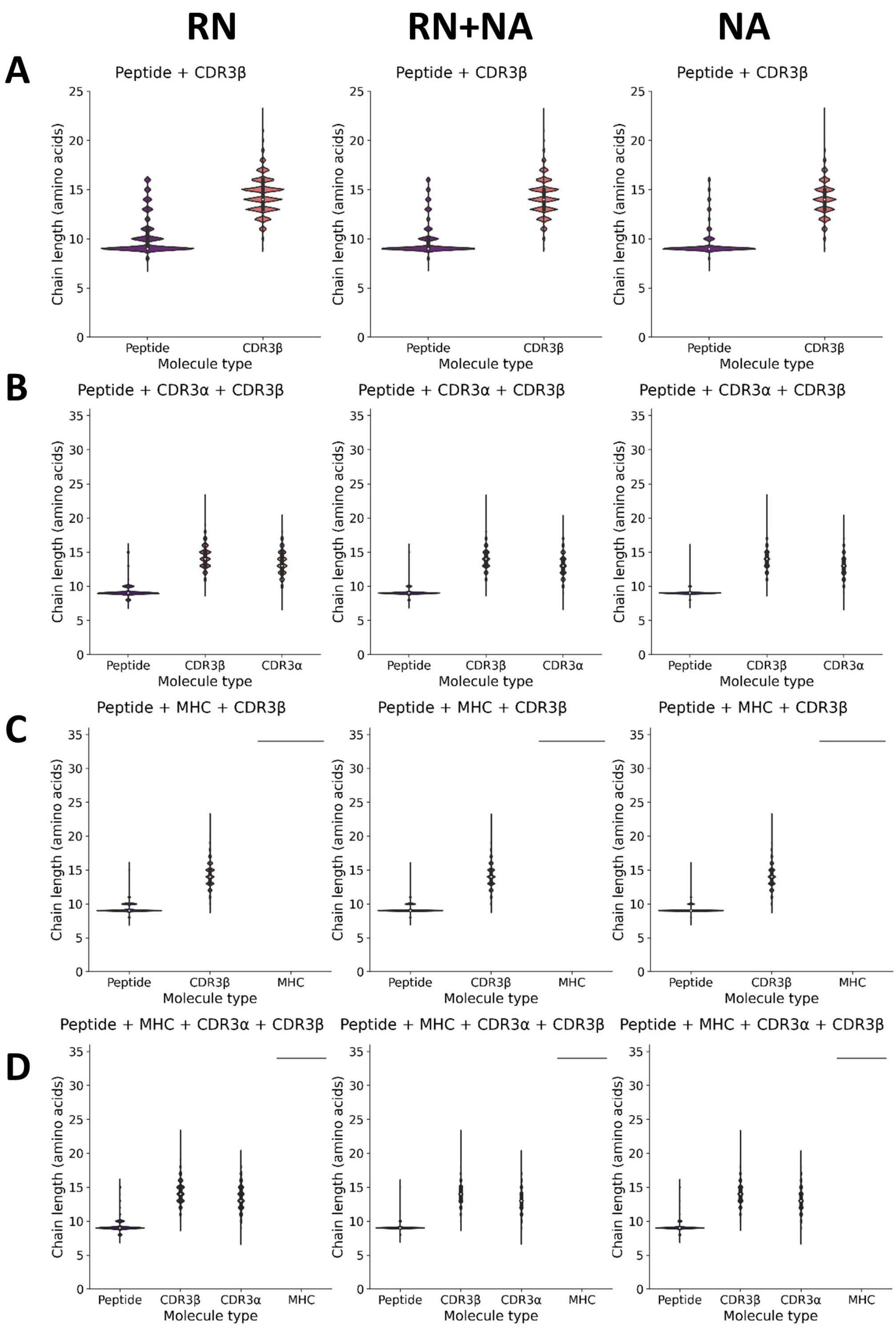

Figure S5: Length distribution of the amino acid sequences for all molecule types. NA: negative samples from negative assays. RN: negative samples from randomized mismatching. **(A)** Length distribution for samples which present $(peptide, CDR3\beta)$. **(B)** Length distribution for samples which present $(peptide, CDR3\beta, CDR3\alpha)$. **(C)** Length distribution for samples which present $(peptide, CDR3\beta, MHC)$. **(D)** Length distribution for samples which present $(peptide, CDR3\beta, CDR3\alpha, MHC)$.

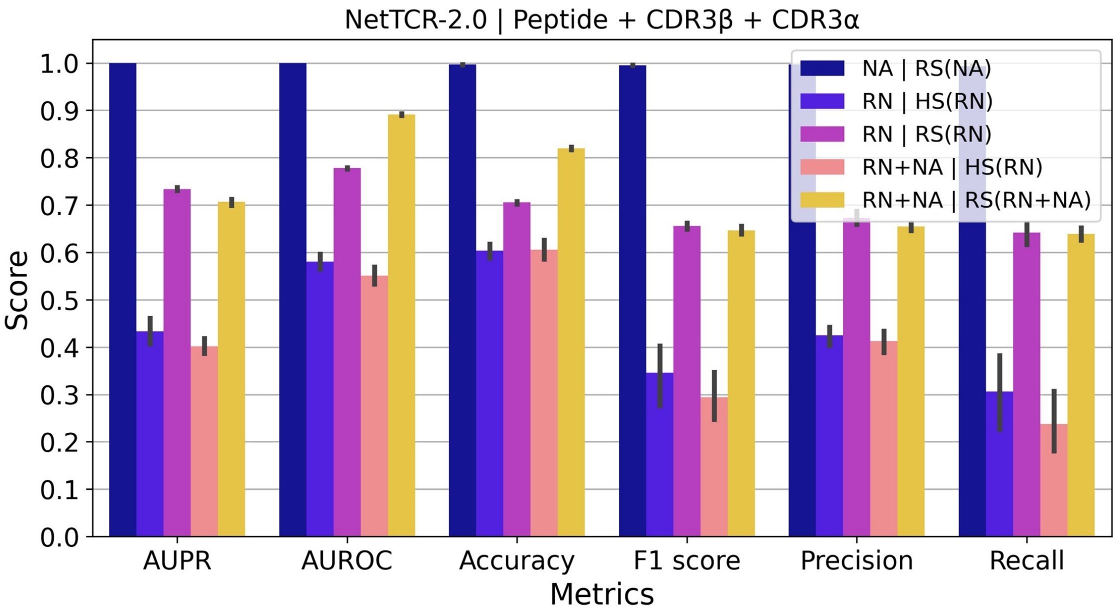

Figure S6: Test results for NetTCR-2.0 for TCR-peptide interaction prediction trained and tested on *TChard*. $(peptide, CDR3\beta, CDR3\alpha, MHC)$ samples are employed, but the MHC is not input to the model. AUPR: area under the precision-recall curve. AUROC: area under the receiver operator characteristic curve. NA: negative samples from negative assays. RN: negative samples from random mismatching. RS(·): random split. HS(·): hard split. Confidence intervals are standard deviation over 5 experiments with independent training/test splits. Legend: *Source of training negatives* | *Training/test split*.

1.2 Tables

Negative samples	Class	(pep, β)	(pep, β, MHC)	(pep, β, α)	$(pep, \beta, \alpha, MHC)$
RN	binding	142,244	100,229	28,410	28,229
	non-binding	259,171	174,311	37,061	36,452
	total	401,415	274,540	65,471	64,681
NA	binding	142,244	100,229	28,410	28,229
	non-binding	126,717	125,969	45,570	45,585
	total	268,961	226,198	73,980	73,814
RN + NA	binding	142,244	100,229	28,410	28,229
	non-binding	385,776	300,168	82,631	82,037
	total	528,020	400,397	111,041	110,266

Table S1. Number of unique tuples (for various combinations of available sequences) in the TChard dataset. RN includes non-binding samples only obtained by random mismatching. NA includes non-binding samples for assays. RN + NA includes non-binding samples from both random mismatching and assays.

Split	Test peptides (positive samples, negative samples)
0	CINGVCWTV (186,385), CRVLCCYVL (435,859), EIYKRWII (180,359), FRCPRRFCF (266,529), FTISVTTEIL (198,395), FVDGVPFVV (2705,5093), GDAALALLLLDRLNQL (609,1191), GTSGSPIINR (173,345), ITEEVGHTDLMAAY (180,354), KAYNVTQAF (807,1579), KLSYGIATV (2458,4643), KPLEFGATSAAL (362,713), KRWIILGLNK (401,837), LITLATCELYHYQECV (251,499), LSPRWYFYYL (1751,3376), NRDVDTDFVNEFYAY (285,566), RAKFKQLL (996,3036), RLRAEAQVK (464,953), RPHERNGFTVL (207,414), SELVIGAVIL (900,1731), SEVGPEHSLAEY (270,534), SPFHPLADNKFAL (248,492), STLPETAVVRR (924,1802), TTDPSFLGRY (244,483), VLPPLLTDEMIAQYT (674,1325), VLWAHGFEL (731,1446), VPHVGEIPVAYRKVLL (528,1042), YEDFLEYHDVRVVL (874,1689), YFPLQSYGF (398,786), YIFFASFYY (353,703), YLNTLTLAV (432,860), YLQPRTFLL (687,1433)
1	LRKVPTDNYITTY (346,682), APKEIIFLEGETL (1783,3356), AYKTFPPTEPK (337,663), CINGVCWTV (186,385), CTFEYVSQPFLM (196,389), EAAGIGILTV (505,1021), ELAGIGILTV (2074,4066), FIAGLIAIV (204,407), FLPFFSNVTWFHAI (299,592), FLPRVFSAV (867,1704), FPPTSFGPL (681,1348), FVDGVPFVV (2705,5093), GMEVTPSGTWLTY (995,1924), ILGLPTQTV (236,468), KAFSPEVIPMF (253,595), KLNVGDYFV (169,337), KLSYGIATV (2458,4643), NPLLYDANYFLCW (548,1069), RNPANNAAIVL (311,615), RSVASQSIIAYTMSL (469,917), SNEKQEILGTVSWNL (451,891), SYFIASFRLFA (219,437), TLIGDCATV (568,1127), VLHSYFTSDYYQLY (483,955), VLPFNDGVYFASTEK (1297,2487), VLWAHGFEL (731,1446), VQELYSPIFLIV (1063,2057), YTMADLVYAL (216,430)
2	APHGVVFLHVTYV (244,484), AVFDRKSDAK (1967,3604), FGEVFNATRFASVY (418,822), FRCPRRFCF (266,529), FTISVTTEIL (198,395), IQYIDIGNY (169,336), KAFSPEVIPMF (253,595), KLPDDFTGCV (1319,2568), KLSYGIATV (2458,4643), KRWIILGLNK (401,837), LEPLVDLPI (417,824), LITLATCELYHYQECV (251,499), LLLDDFVEII (968,1864), LSPRWYFYYL (1751,3376), LVVDFSQFSR (1871,3600), MGYINVFAFPFTIYSL (2918,5236), RNPANNAAIVL (311,615), SEVGPEHSLAEY (270,534), SNEKQEILGTVSWNL (451,891), TLIGDCATV (568,1127), TPRVTGGGAM (2557,4778), TVLSFCAFAV (613,1202), VLPPLLTDEMIAQYT (674,1325), YFPLQSYGF (398,786)
3	AELAKNVSLDNVL (1794,3382), ALSKGVHFV (170,340), AMFWSVPTV (182,363), APHGVVFLHVTYV (244,484), EAAGIGILTV (505,1021), FLCLFLLPSLATV (244,483), FPPTSFGPL (681,1348), FTISVTTEIL (198,395), FVDGVPFVV (2705,5093), GMEVTPSGTWLTY (995,1924), GNYTVSCLPFTI (176,351), ILGLPTQTV (236,468), ITEEVGHTDLMAAY (180,354), KTAYSHLSTSK (474,936), LEPLVDLPI (417,824), LLLDDFVEII (968,1864), LVVDFSQFSR (1871,3600), NPLLYDANYFLCW (548,1069), SEHDYQIGGYTEKW (3424,6114), SPFHPLADNKFAL (248,492), STLPETAVVRR (924,1802), TPINLVRDL (266,528), TPRVTGGGAM (2557,4778), TVLSFCAFAV (613,1202), YLDAYNMMI (221,439)
4	AELAKNVSLDNVL (1794,3382), ALRKVPTDNYITTY (346,682), APHGVVFLHVTYV (244,484), CRVLCCYVL (435,859), GDAALALLLLDRLNQL (609,1191), GMEVTPSGTWLTY (995,1924), KAYNVTQAF (807,1579), KLSYGIATV (2458,4643), LEPLVDLPI (417,824), LITGRLQSLQTYV (261,518), LITLATCELYHYQECV (251,499), LLWNGPMAV (2559,4939), MGYINVFAFPFTIYSL (2918,5236), MPASWVMRI (777,1522), PKYVKQNTLKLAT (412,1316), QLMCQPILLL (980,1912), RFYKTLRAEQASQ (282,569), RLRAEAQVK (464,953), RNPANNAAIVL (311,615), SEVGPEHSLAEY (270,534), SFHSLHLLF (186,371), SPRWYFYYL (214,425), STLPETAVVRR (924,1802), VPHVGEIPVAYRKVLL (528,1042), VTEHDTLLY (275,543), YEQYIKWPWYI (537,1057), YYVGYLQPRTFLL (365,721)

Table S2. Hard splits for $(peptide, CDR3\beta)$ samples. The numbers enclosed in parentheses refer to the positive (binding) and negative (non-binding) samples, respectively, which present a given peptide.

Split	Test peptides
0	AVFDRKSDAK (1852,3009), DATYQRTRALVR (100,200), EAAGIGILTV (42,84), FEDLRVSSF (34,68), FLRGRAYGL (43,86), IVTDFSVIK (747,1332), LLWNGPMAV (671,1306), NLVPMVATV (348,677), PKYVKQNTLKLAT (62,124), RPRGEVRFL (116,232), RTLNAWVKV (51,102)
1	AVFDRKSDAK (1852,3009), CINGVCWTV (84,166), EAAGIGILTV (42,84), FEDLRVLSF (45,90), FLASKIGRLV (35,70), FLCMKALLL (136,270), FLRGRAYGL (43,86), FLYALALLL (39,78), FTSDYYQLY (38,76), KLSALGINAV (45,90), KLVALGINAV (66,132), LLWNGPMAV (671,1306), LTDEMIAQY (131,261), RLRAEAQVK (442,832), VLFGLGFAI (35,69), VVMSWAPPV (41,82), YVLDHLIVV (141,281)
2	CTELKLSDY (61,122), EAAGIGILTV (42,84), ELAGIGILTV (530,1028), FEDLRLLSF (43,86), FEDLRVLSF (45,90), FLASKIGRLV (35,70), FLCMKALLL (136,270), FLRGRAYGL (43,86), FTSDYYQLY (38,76), GLCTLVAML (399,761), KLVALGINAV (66,132), KMVAVFYTT (42,83), LTDEMIAQY (131,261), NYNYLYRLF (35,70), PKYVKQNTLKLAT (62,124), RAKFKQLL (1268,2297), SPRWYFYYL (142,281), VLFGLGFAI (35,69), YLQPRTFLL (267,528), YVLDHLIVV (141,281)
3	AVFDRKSDAK (1852,3009), CTELKLSDY (61,122), FEDLRVSSF (34,68), FLYALALLL (39,78), FTSDYYQLY (38,76), IVTDFSVIK (747,1332), LLFGYPVYV (74,149), LTDEMIAQY (131,261), NLNCCSVPV (54,108), NYNYLYRLF (35,70), PKYVKQNTLKLAT (62,124), YLQPRTFLL (267,528), YVLDHLIVV (141,281)
4	AVFDRKSDAK (1852,3009), AYAQKIFKI (39,77), DATYQRTRALVR (100,200), EAAGIGILTV (42,84), ELAGIGILTV (530,1028), FLASKIGRLV (35,70), FLCMKALLL (136,270), FLRGRAYGL (43,86), FLYALALLL (39,78), GLCTLVAML (399,761), NLNCCSVPV (54,108), NYNYLYRLF (35,70), PKYVKQNTLKLAT (62,124), SPRWYFYYL (142,281), VLFGLGFAI (35,69), YVLDHLIVV (141,281)

Table S3. Hard splits for $(peptide, CDR3\beta, CDR3\alpha, MHC)$ samples. The numbers enclosed in parentheses refer to the positive (binding) and negative (non-binding) samples, respectively, which present a given peptide.

2 TRAINING/TEST SPLITTING STRATEGIES IN RELATED WORKS

In this section, we describe how ERGO II and NetTCR-2.0 perform training/test splitting and how this differs from our approach.

Springer et al. (2021) propose four different settings. In the Single Peptide Binding (SPB) setting, it is tested whether an unknown TCR binds to a predefined target peptide; at training time, TCRs which are known to bind to that peptide are employed. In the TCR-Peptide Pairing I (TPP-I) setting, which is comparable to our RS, test peptides and TCRs can be observed at training time. In the TCR-Peptide Pairing II (TPP-II) setting, test TCRs cannot be observed at training time, but peptides can. In the TCR-Peptide Pairing III (TPP-III) setting, it is ensured that both test TCRs and test peptides are unseen, i.e. not included in training tuples. Mismatched negative samples are derived from a randomization heuristic, analogous to how we construct the non-binding set in this work (see Section The *TChard* dataset).

Montemurro et al. (2021) compute the peptide-specific Levenshtein distance among CDR3s. Using the Hobohm 1 algorithm Hobohm et al. (1992), redundancies among the CDR3s are removed. Five partitions are created to allow cross-validation. Single-linkage clustering of the redundancy-reduced positive training data is performed for partitioning and negative samples from 10X Genomics and randomization are added. For evaluating the model, test data points are separated from the training data by a given Levenshtein similarity threshold, i.e. samples with similarities to the training data above this threshold were removed. In contrast to our work, Montemurro et al. (2021) do not investigate generalization on unseen peptides.

3 INVALID RESIDUES AND CDR3 SEQUENCE HOMOGENIZATION

We highlight that certain amino acid sequences present the symbols ’X’ and ’O’ for unknown residues; we do not remove those sequences. Furthermore, the CDR3 sequences do not present, in general, the same format across the various source databases. Certain sequences start e.g. with ‘CASS’, while others omit the initial ‘C’. We homogenize the CDR3 sequences while pre-processing the dataset adding the initial ‘C’, when missing.

4 RESULTS ON HIGHER QUALITY VDJDB SAMPLES

In this section, we describe experiments performed on a subset of VDJdb obtained by excluding the low-quality samples (score = 0). For these experiments, we removed the rows of the VDJdb dataframe which present a score of 0; only samples with scores 1, 2 or 3 are included in the analysis. The data pre-processing follows the exact same steps described in the main text. No length filtration is performed on the sequences, due to the negligible number of outliers. Due to the smaller amount of samples compared to TChard, we only conduct the experiment in the peptide+CDR3 setting (3453 binding pairs, 3278 CDR3, 266 epitopes). We derive the negative pairs via randomization and operate the hard split (HS) setting $l = 10$ and $u = 100$ (see Table S4).

Figure S7A depicts the class distribution of pepides and CDR3 sequences. Figure S7B depicts their length distribution. Experimental results are shown in Figure S7C for both ERGO II and NetTCR-2.0. Both model fail to generalize to unseen test epitopes. These results, computed exclusively on VDJdb excluding the low quality samples, confirm the results we obtained on the whole TChard dataset.

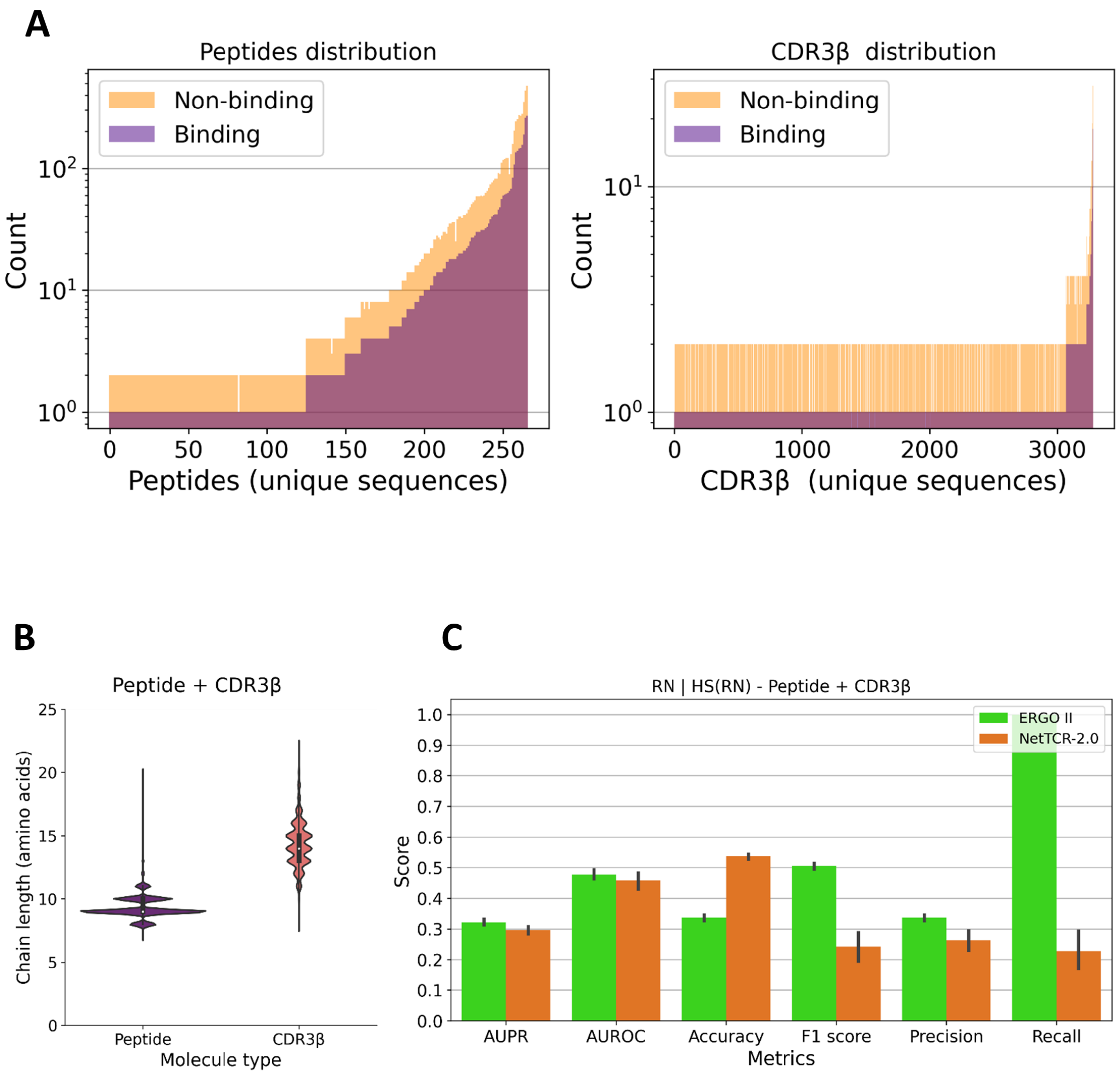

Figure S7: All figures refer to the set of experiments performed on the VDJdb samples with score $\geq$ 1. (A) Separate class distributions for unique peptide and CDR3β sequences in all $(peptide, CDR3\beta)$ samples. (B) Length distributions. (C) ERGO II and NetTCR-2.0 results in the peptide+CDR3β setting using the hard split (HS). Confidence intervals are standard deviation over 5 experiments with independent training/test hard splits. All negative samples are random negatives (RN), i.e. derived from randomization.

Split	Test peptides (positive samples, negative samples)
0	AAFKRSCLK (5,10), APRGPHGGAASGL (5,10), CRVLCCYVL (31,60), CTELKLSDY (4,8), EAAGIGILTV (27,52), FLKETGGL (4,7), FLRGRAYGL (15,33), FPRPWLHGL (30,59), FPTKDVAL (10,20), FYGKTILWF (4,8), GPGMKARVL (4,8), GTSGSPIIDK (19,38), HPKVSSEVHI (25,48), HPVGEADYFEY (24,59), ISPRTLNAW (22,43), KLSALGINAV (5,10), LLWNGPMAV (18,36), LPPIVAKEI (20,40), LPRRSGAAGA (7,14), MLNIPSINV (27,51), NLSALGIFST (18,36), QIKVRVDMV (7,14), QYDPVAALF (11,22), RPHERNGFTVL (22,46), RPPIFIRRL (28,55), SLLMWITQV (5,10), SPRWYFYYL (14,30), TAFTIPSI (13,34), TPGPGVRYPL (33,65), VAANIVLTV (14,27), VTEHDTLLY (10,20), VVMSWAPPV (8,15), YLEPGPVTA (4,8), YSEHPTFTSQY (20,40)
1	AAGIGILTV (5,10), APRGPHGGAASGL (5,10), ARMILMTHF (14,26), CLGGLLTMV (4,8), CRVLCCYVL (31,60), DATYQRTRALVR (27,59), EAAGIGILTV (27,52), EPLPQGQLTAY (28,69), FLGKIWPSHK (8,16), FLKETGGL (4,7), FPRPWLHGL (30,59), FPTKDVAL (10,20), FYGKTILWF (4,8), GLNKIVRMY (13,26), HPVGEADYFEY (24,59), ILKEPVHGV (7,14), ISPRTLNAW (22,43), IVTDFSVIK (21,41), KASEKIFYV (4,8), KRWIIMGLNK (31,60), LLWNGPMAV (18,36), LPPIVAKEI (20,40), NLNCCSVPV (4,10), QASQEVKNW (8,16), RLRPGGKKR (13,26), RLRPGGRKR (6,12), SLLMWITQV (5,10), SLYNTVATL (32,63), TLNAWVKVV (4,8), TPGPGVRYPL (33,65), VSFIEFVGW (14,27), YLEPGPVTA (4,8), YPLHEQHGM (9,18), YSEHPTFTSQY (20,40)
2	ALDPHSGHFV (4,8), ALTPVVVTL (6,12), ALYGFVPVL (6,12), ARMILMTHF (14,26), AVFDRKSDAK (9,18), CLGGLLTMV (4,8), CTELKLSDY (4,8), CVNGSCFTV (14,25), EPLPQGQLTAY (28,69), FLKEMGGL (4,8), FLKEQGGL (4,8), FLRGRAYGL (15,33), FPTKDVAL (10,20), FRCPRRFCF (10,20), GLNKIVRMY (13,26), GTSGSPIIDK (19,38), HMTEVVRHC (4,10), HPVGEADYFEY (24,59), HSKKKCDEL (30,59), ILKEPVHGV (7,14), KASEKIFYV (4,8), LLLGIGILV (9,17), LLWNGPMAV (18,36), LPRRSGAAGA (7,14), MTLHGFMMY (4,8), NLNCCSVPV (4,10), NLSALGIFST (18,36), NYNYLYRLF (13,26), QASQEVKNW (8,16), QIKVRVDMV (7,14), QIKVRVKMV (11,22), QYDPVAALF (11,22), QYIKWPWYI (18,21), RLARLALVL (7,14), RLRPGGKKK (20,39), RLRPGGRKR (6,12), SPRWYFYYL (14,30), TPGPGVRYPL (33,65), VAANIVLTV (14,27), VSFIEFVGW (14,27), VVMSWAPPV (8,15), YPLHEQHGM (9,18), YSEHPTFTSQY (20,40)
3	AAFKRSCLK (5,10), ALDPHSGHFV (4,8), ALTPVVVTL (6,12), ALYGFVPVL (6,12), APRGPHGGAASGL (5,10), CTELKLSDY (4,8), DATYQRTRALVR (27,59), ELRRKMMYM (4,8), FLKEMGGL (4,8), FPRPWLHGL (30,59), FRCPRRFCF (10,20), FYGKTILWF (4,8), GLNKIVRMY (13,26), GPGMKARVL (4,8), GTSGSPIIDK (19,38), HMTEVVRHC (4,10), HPVGEADYFEY (24,59), ISPRTLNAW (22,43), KASEKIFYV (4,8), KRWIIMGLNK (31,60), LLLGIGILV (9,17), LLWNGPMAV (18,36), MLNIPSINV (27,51), MTLHGFMMY (4,8), NLNCCSVPV (4,10), NYNYLYRLF (13,26), QIKVRVKMV (11,22), QYIKWPWYI (18,21), RLQSLQTYV (21,41), RLRPGGKKK (20,39), RLRPGGKKR (13,26), RLRPGGRKR (6,12), RPHERNGFTV (4,7), SLYNTVATL (32,63), SPRWYFYYL (14,30), TLNAWVKVV (4,8), TPGPGVRYPL (33,65), VLEETSVML (14,28), VTEHDTLLY (10,20), YPLHEQHGM (9,18)
4	AAFKRSCLK (5,10), AAGIGILTV (5,10), ALYGFVPVL (6,12), APRGPHGGAASGL (5,10), ARMILMTHF (14,26), CLGGLLTMV (4,8), CTELKLSDY (4,8), EAAGIGILTV (27,52), ELRRKMMYM (4,8), FLKEQGGL (4,8), FYGKTILWF (4,8), GADGVGKSAL (5,9), GLNKIVRMY (13,26), HMTEVVRHC (4,10), HPKVSSEVHI (25,48), IIKDYGKQM (18,35), KLSALGINAV (5,10), KRWIIMGLNK (31,60), LLWNGPMAV (18,36), LPEPLPQGQLTAY (5,14), LPRRSGAAGA (7,14), MTLHGFMMY (4,8), NLNCCSVPV (4,10), NLSALGIFST (18,36), NYNYLYRLF (13,26), QASQEVKNW (8,16), QIKVRVDMV (7,14), QIKVRVKMV (11,22), QYDPVAALF (11,22), RLARLALVL (7,14), RLQSLQTYV (21,41), RLRPGGKKR (13,26), RMFPNAPYL (4,8), RPHERNGFTV (4,7), RPPIFIRRL (28,55), SLLMWITQV (5,10), SLYNTVATL (32,63), SPRWYFYYL (14,30), TAFTIPSI (13,34), TLNAWVKVV (4,8), VAANIVLTV (14,27), VLEETSVML (14,28), VQIISCQY (2,8), VVMSWAPPV (8,15), VYALIAGATL (4,8), YPLHEQHGM (9,18), YSEHPTFTSQY (20,40)

Table S4. Hard splits for $(peptide, CDR3\beta)$ derived from the VDJdb samples with score ≥ 1. The numbers enclosed in parentheses refer to the positive (binding) and negative (non-binding) samples, respectively, which present a given peptide.

REFERENCES

Springer I, Tickotsky N, Louzoun Y. Contribution of t cell receptor alpha and beta cdr3, mhc typing, v and j genes to peptide binding prediction. *Frontiers in immunology* **12** (2021).

Montemurro A, Schuster V, Povlsen HR, Bentzen AK, Jurtz V, Chronister WD, et al. Nettcr-2.0 enables accurate prediction of tcr-peptide binding by using paired tcrα and β sequence data. *Communications biology* **4** (2021) 1–13.

Hobohm U, Scharf M, Schneider R, Sander C. Selection of representative protein data sets. *Protein Science* **1** (1992) 409–417.
